# Developing a Framework for a Program Theory-Based Approach to Evaluating Policy Processes and Outcomes: Health in All Policies in South Australia

**DOI:** 10.15171/ijhpm.2017.121

**Published:** 2017-10-22

**Authors:** Angela Lawless, Fran Baum, Toni Delany-Crowe, Colin MacDougall, Carmel Williams, Dennis McDermott, Helen van Eyk

**Affiliations:** ^1^College of Nursing and Health Sciences, Flinders University, Adelaide, SA, Australia.; ^2^Southgate Institute for Health Society and Equity, Flinders University, Adelaide, SA, Australia.; ^3^College of Medicine and Public Health, Flinders University, Adelaide, SA, Australia.; ^4^Department of Health and Ageing, Adelaide, SA, Australia.; ^5^The Poche Centre for Indigenous Health and Well-being, Flinders University, Adelaide, SA, Australia.

**Keywords:** Healthy Public Policy, Evaluation, Inter-sectoral Action, Health Equity, Social Determinants

## Abstract

**Background:** The importance of evaluating policy processes to achieve health equity is well recognised but such evaluation encounters methodological, theoretical and political challenges. This paper describes how a program theorybased evaluation framework can be developed and tested, using the example of an evaluation of the South Australian Health in All Policies (HiAP) initiative.

**Methods:** A framework of the theorised components and relationships of the HiAP initiative was produced to guide evaluation. The framework was the product of a collaborative, iterative process underpinned by a policy-research partnership and drew on social and political science theory and relevant policy literature.

**Results:** The process engaged key stakeholders to capture both HiAP specific and broader bureaucratic knowledge and was informed by a number of social and political science theories. The framework provides a basis for exploring the interactions between framework components and how they shape policy-making and public policy. It also enables an assessment of HiAP’s success in integrating health and equity considerations in policies, thereby laying a foundation for predicting the impacts of resulting policies.

**Conclusion:** The use of a program theory-based evaluation framework developed through a consultative process and informed by social and political science theory has accommodated the complexity of public policy-making. The framework allows for examination of HiAP processes and impacts, and for the tracking of contribution towards distal outcomes through the explicit articulation of the underpinning program theory.

Since 2008 the South Australian Government has implemented a Health in All Policies (HiAP) approach to facilitate the development of healthy public policy. A five year action research program was established to examine the changes in policy-making processes as a result of the HiAP initiative, and to assess the impacts of these processes. This paper examines the development of a program theory-based framework used to guide evaluation of the South Australian HiAP initiative over a five year period.

## Background

Evaluation of policy-making processes and outcomes presents methodological, theoretical and political challenges.^1,2^ Policy and policy work is understood and described in diverse and sometimes conflicting ways^3^ but these ‘multiple accounts’ can help make sense of the processes, each adding a particular perspective.^2,4^ Such interpretive approaches to policy-making suggest that research should examine how institutions shape thinking and working, attend to the role of relationships and boundaries in the ‘doing’ of policy work, uncover the actual practices of policy-making and the role of ideas and ideation processes in reform efforts, problem identification and representation.^4-6^ The activities of policy-making are broad and include the identification and conceptualisation of problems, gaining the attention of government, the formulation of policy alternatives, selection of policy solutions and implementation, evaluation and revision – not as discrete episodes but as an ongoing ‘juggling’ process.^2,3^ Evaluators, as well as the policy-makers need to negotiate the political context which includes political cycles, key personnel changes and the waxing and waning of political support.^7^ Practically there may be obstacles in gaining access to the diverse individuals and groups involved in policy-making as researchers encounter gatekeepers and seek to engage time-poor bureaucrats and politicians.^2,8,9^ Program theory-based evaluation offers a means of evaluation that engages with these difficulties in a rigorous and systematic manner.^10^

Program theory has been defined as “the process through which program components are presumed to affect outcomes and the conditions under which these processes are believed to operate.”^10^ By uncovering the theories underlying policy-making processes, explicit links and pathways can be hypothesized between program components and outcomes. Program theory can also include identification of the assumptions that underpin an initiative and the risks to those assumptions^11^ as well as how the initiative relates to the economic, social and political environments.

It is well understood that this broader context fundamentally shapes interventions but it is often not well accounted for in evaluation.^12^ Consideration of context is essential in uncovering the reasons and conditions in which a particular intervention works and how well it works.^13^ Disappointing results or failure may be the result of factors external to the program itself. Real-life policy-making is driven by a wide range of contextual factors, including power relations, which make it a ‘messy’ undertaking.^3^ Politics, layers of administration, and non-government organisations all form part of the context of policy processes.^14^ An exploration of possible implications of contextual factors for the program is central to development and testing of program theory. Context is also central to the success or otherwise of programs becoming embedded and routinized.^12^ In constantly changing policy contexts adaptability of interventions is a key to their success.^15,16^

Where outcomes are long-term, theory-based evaluation allows identification of interim indicators of progress toward those long-term outcomes. Such evaluations provide a means of elaborating program theory and provide a framework for decisions about which aspects of the program will be measured or evaluated. Evaluation can thus be structured to examine the theories thought to underpin the program, and data collection designed to permit investigation of and confirm that theory.^17^ The program theory underpinning a program such as HiAP is developed and then the key activities and events of the program are examined through multiple data sources in various contexts (eg, in various instances of health lens analysis [HLA]) allowing testing of the program theory.

Whilst interim steps in the pathway between an intervention and the achievement of high level outcomes can be identified, establishing causality is particularly difficult in complex health initiatives. Contribution analysis offers a step-wise, structured approach to the examination and testing of causal claims.^18^ “[Contribution Analysis] assesses causal chains from beginning to end, reports on whether the intended changes occurred or not, and identifies the main contributions to such changes, including the intervention under evaluation” (p. 281).^19^ Where a causal chain can be verified with empirical evidence and other major external influencing factors accounted for, then credible conclusions can be made regarding the contribution of an intervention to specified outcomes. Policy-making entails many complex elements and interactions, therefore accounting for all the external influences affecting the outcomes may prove impractical if not impossible. A pragmatic but systematic approach to causal analysis - which tests the congruence of the program theory with the results, employs counterfactual comparisons where possible, reviews results critically and seeks to explain exceptions and identify alternative explanations - may be more appropriate.^20^

Key actors can assist researchers in building a robust understanding of an intervention, its causal pathways and its context. Such involvement requires the engagement of policy actors in making sense of the data strategically and operationally. As well as knowledge about the specific intervention, policy actors also contribute broader bureaucratic knowledge - the experiential and tacit knowledge of processes, contexts and audiences, and perceptions of what constitutes viable policy options.^21^ Colebatch maintains that ‘getting close’ to policy processes and policy actors is particularly important for research to trace the ‘evolution of policy work over time.’^6^

### Health in All Policies in South Australia

The process followed by the South Australian Government in developing and implementing HiAP is described in Box 1. The adoption of HiAP by the South Australian Government provides an opportunity to examine the “juggling” of resources and negotiation of barriers undertaken by policy actors in an authentic context, that is, where complex interactions between actors, strategies and institutions play out in attempts to deliver better government.^22^

**Box 1. Health in All Policies in South Australia**

In 2008 the South Australian Government committed to the adoption of a HiAP approach. Considerable work had been undertaken to prepare the way for such an approach and, as with the European initiative, HiAP in South Australian built on a significant history of advocacy and innovation regarding healthy public policy. By 2010 a dedicated HiAP unit had been established within the Health department, governance for the initiative was linked to processes already in place for the State’s strategic plan^23^ and a process termed ‘health lens analysis’ had been developed and implemented in collaboration with a number of sectors.^24^

**Figure:**
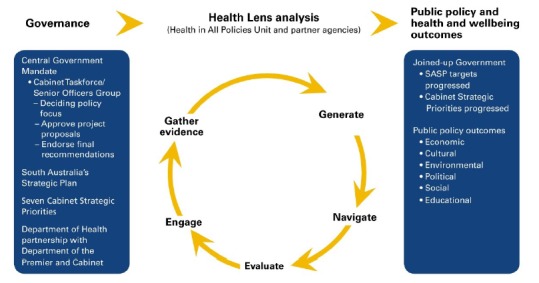
Source: The South Australian Model of Health in All Policies 2011 (p. 26).25

HiAP is based on the understanding that health is shaped by social, economic and environmental determinants, and that action to improve health requires the involvement of multiple sectors and actors.^26^ HiAP has been described as an approach that seeks to use “all available measures in all policy fields” (p. xvi)^27^ and is designed to encourage action to improve population health and health equity across government sectors.^26^ It involves multiple partners with sometimes divergent agendas and is implemented in, and influenced by, changing political, organisational and economic contexts.^28^ It requires negotiation of relationships, values and meaning across organisational boundaries.^14^

HiAP has been characterised as an “instrumental process-based intervention” – instrumental in its potential to create healthier policy through a focus on government processes.^5,29^ Carey et al suggest that HiAP is a “set of institutional arrangements for delivering JUG (joined up government)” which in the South Australian case operates within the Government bureaucracy in a top-down manner.^29^ These institutional policy-making arrangements structure the decision-making and frame the policy action (and inaction).^22^

Understanding the policy-making processes and contexts that support consideration of health and equity in the policies of non-health sectors is critical to the success and maintenance of HiAP. This paper describes development of a framework that has guided the evaluation of the South Australian HiAP initiative and discusses its benefits and limitations.

## Methods: How the Evaluation Framework Was Developed

The evaluation approach described in this paper is rooted in the tradition of theory-based approaches to complex community-based initiatives (see for example^30,31^). Whilst there are differences between theory-based approaches, Stame identifies four similarities that span theory-based approaches:

- The evaluation is based on an account of what is thought likely to happen as a result of the initiative.
- They take account of context.
- The approach utilises a range of methods.
- They are committed to internal validity and to looking for causal relationships.^32^

Theory-based evaluation makes the causal assumptions behind policy interventions explicit, ie, it explains how and why a program or policy is thought to work, which forms the logic that underpins an initiative.^33^ As Leeuw and others note, program theory is often drawn from stakeholder knowledge and is considered distinct from substantive social science theory, which may nevertheless inform and enrich program theory.^30,33^ A distinction can also be drawn between program theory and implementation theory.^31,34,35^ Program theory is concerned with mechanisms leading to the desired changes rather than the activities per se.^35^ Implementation theory sheds light on how a particular initiative is operating, and program theory seeks to understand how program effects are realized.^17^

To develop the framework that is presented in this paper, the research team undertook an iterative, participatory process to develop and refine an evaluation framework. Analysis of South Australia (SA) Health in All Policies Unit documents was undertaken, involving examination of approximately 300 sources, including policy documents, HiAP project proposals, project reports, online materials, training materials and a book. The documents were analysed to determine how HiAP was described, what rationales for HiAP were given, what activities were being undertaken and what outcomes were hoped to be achieved.

Two research workshops were held to capture the ‘history, experience and intuition of key stakeholders.’^36^ The research team had considerable experience in use of theory-based evaluation and had used such workshops previously to explicate program theory.^37^ They adapted the method from their previous experience to apply to HiAP in SA. The purpose of these workshops was to elaborate how HiAP was operationalised in SA and examine how and why HiAP was being implemented in a particular way and what outcomes were expected. The study employed theoretical sampling in seeking informants with specific experience in either the development of HiAP in SA and/or implementation of HiAP. Involving stakeholders in the articulation of program theory strengthens the utility, feasibility, propriety and accuracy of the study.^30^ The first workshop was attended by 9 public servants (policy officers, middle managers and a director) from the education, planning, transport, local government, rural health and mining sectors of the SA Government. Participants had all been directly involved in a HiAP HLA project. The second workshop involved 16 employees from the SA Department of Health (policy officers, middle managers and directors) with specific knowledge of, and responsibilities for, HiAP. These participants included those who had been involved in the development of HiAP in SA as well as those who had participated in HLA projects.

During the workshops, participants also contributed bureaucratic knowledge^21^ about administrative procedures and processes and the political milieu. The research team contributed knowledge of the initiative gained through observation and interaction with the HiAP initiative and formative evaluation of HiAP projects,^24^ as well as insights from research regarding healthy public policy and inter-sectoral action. As Birckmayer and Weiss suggest, the researchers “cycled through” the various sources and synthesized the information to construct an agreed upon program theory.^38^

At the stage of framework development, agenda setting theory provided by Kingdon was considered to be a useful theoretical lens for analysing the HiAP initiatives because it explains how evidence, theory and political processes respond to a range of influences – ideas, interests and institutions. A key characteristic of the SA case has been the early and proactive engagement of health with other sectors *prior* to a particular policy proposal being suggested. This early engagement means that HiAP can contribute to the agenda-setting phase of the policy process^39^ which was seen as an important point of difference between the SA model and health impact assessment.^40^ The utility of Kingdon’s framework in relation to HiAP has been recognized by others^41,42^ and is especially pertinent to explanations of how and why the social determinants of health rarely reach the policy agenda^43^ He proposes that policy action requires a ‘policy window’ to become available through linkage of three streams: problems, policy and politics.

The Kingdon framework informed the themes explored in the research workshops, with participants asked to consider the ways in which HiAP was able to establish itself on the agenda of multiple sectors and its strengths and weaknesses as a process of policy reform to enable action on determinants of health.

Each four hour workshop included an introduction to program theory-based modelling. Broad questions exploring the impetus and start-up phase of HiAP were posed. These included the conditions that facilitated or hindered the Government’s adoption and implementation of the approach. A guided discussion then drew out the assumptions underpinning the HiAP approach, its activities and processes and the shorter-term changes and long-term outcomes expected. Workshop proceedings were audio recorded and transcribed verbatim. The research team conducted a collaborative, thematic analysis of the transcripts using NVivo 10. Following an initial open coding, selective coding^44^ was applied to the transcripts to explore the participants’ discussion of HiAP and the contextual factors, assumptions, activities and outcomes that they associate with it. This analysis, together with knowledge and insights from relevant literature and HiAP documentation was used to develop a framework of the South Australian HiAP initiative.

After the workshops, the draft framework was circulated to participants by email inviting comment and clarification. Seven participants took the opportunity to provide feedback. Feedback was considered by the research team, modifications made and the revised framework was re-circulated. The framework was discussed and endorsed at a meeting of the Project Advisory Group comprising project investigators and key stakeholders (including representatives from Indigenous health, the social services peak body and the Department of the Premier and Cabinet).

As the framework was applied to guide the subsequent evaluation we drew on other social and political science theories both to identify key questions and better understand the emerging findings. This will be further elaborated in the Discussion section.

## Results: Understanding the HiAP Evaluation Framework

**Figure 1:**
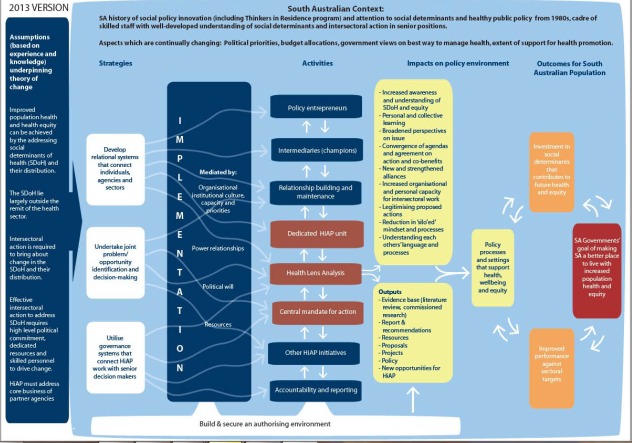
South Australia Health in All Policies Evaluation Framework 2013.

The product of this process was a graphic framework of the South Australian HiAP initiative (see Figure 1). The framework incorporates the components of the HiAP initiative identified through the development process, that is to say HiAP’s strategies, mediating factors influencing its implementation and its activities, the contextual factors that influence HiAP and the theories of change that underpin it. This provides a framework for researchers to seek credible evidence of change at each link in the chain.

The SA HiAP initiative consists of three main strategies that are seen as mutually reinforcing: (1) developing relational systems that connect individuals, agencies and sectors; (2) undertaking joint problem identification and problem-solving; and (3) utilising governance systems that connect HiAP work with senior decision-makers. A range of actors, structures and processes involved in HiAP, were identified as the means through which these strategies were enacted and we have termed these ‘activities.’ These activities are thought to result in a range of intermediate changes, including the way policy actors think about and act on health and equity considerations; the facilitation of “joined-up” policy work through development of shared agendas and common understandings of within-sector processes; the development of new inter-sectoral alliances and strengthening of existing ones; and increased organisational and personal capacity for inter-sectoral work. These changes are considered to create favourable conditions for the development and implementation of policies that support health, wellbeing and equity.

The theory of change is shown through the directional arrows in the model. These arrows provide a visual representation of the theory of change, linking the assumptions that inform HiAP, the strategies that are used, the activities that are generated, the impacts that are produced within and outside of government, and then they link these to the eventual anticipated outcomes for the South Australian population. To maintain readability we have indicated only the strongest links between the strategies and the components by these arrows (for example, ‘develop relational systems that connect individuals, agencies and sectors’ is linked to ‘intermediaries’), but it is recognised that many of the components form part of more than one strategy.

The discussion of results below is structured according to the components of this framework, that is to say the South Australian context, assumptions, strategies and activities, amongst others.

### Context

The workshops explored the conditions that made the adoption of HiAP possible and the contextual factors that continue to shape the initiative. We sought to include the key contextual factors that impact on policy-making and resulting policies. As Pollitt^45^ notes context is difficult to define, theorise and operationalise. Clarke’s idea of contexts animating action provides a useful means of exploring context – what makes “things thinkable (in Foucault’s sense), possible, relevant, desirable and necessary” (p. 24).^46^ In this sense context acts to make particular actions, in this case HiAP, possible as well the actors that enact them.

Workshop participants suggested that the evaluation should examine the interaction between HiAP and factors such as the political priorities of the day, budgetary pressures and prevailing views about health and equity. For example in terms of political priorities SA’s Strategic Plan provided a scaffold for HiAP, linking policy action to an existing framework and providing a ready-made accountability mechanism.^47^

They also emphasised the importance of the legacy of social innovation and social justice in SA. This history was manifest in a cadre of public servants with the knowledge, skills and capacity to advocate for and implement the ideas associated with HiAP. Policy actors’ understanding of these key ideas was considered important in shaping a receptive context for an innovative policy agenda.

“… *in the ‘70s there was quite a culture of working collaboratively and a lot of emphasis on social justice … some of the people that would have been working on the ground at that point had actually gone up high enough in the organisations for that to re-emerge as something that supported [HiAP]….”* (Research workshop 1, non-health sector).

*“…there’s a strong link between the kinds of things we do in HiAP and basic community development approaches… starting in their territory, working with them in partnership; that basic community development stuff that was happening, … and they’re eminently transferable into what is now a more bureaucratic kind of environment… I think in relation to the top down it was important that there were a few ministers and a few chief executives who were sympathetic, broadly, to this approach”* (Research workshop 2, health sector).

Diagrammatically, context is represented as the background space. It is in this space that the HiAP initiative is conceptualised, developed and implemented, and outcomes realised (or not). The limitations of two dimensional representations restrict the extent to which the dynamic and sometimes turbulent interaction between initiative and context can be captured.

### Assumptions

The workshops also explored the assumptions that needed to be included in a framework to explain the rationale underpinning the South Australian HiAP initiative. These assumptions position HiAP as a response to knowledge regarding the social and economic determinants of health and acknowledge that improvements in population health and health equity require policy actions in all sectors that have an impact on health. The need for political commitment and adequate allocation of resources was noted. The cooperative nature of the South Australian HiAP initiative was emphasized - HiAP must address the core business of other sectors, not only the health sector’s agenda:

“…*the need for complementary goals for the participating organisations is a starting point for the approach … If you don’t have that common purpose or complementary purpose you’re not going to get anywhere”* (Research workshop 1, non-health sector).

*“… you could actually present collaboration with health as a positive way…that collaborating with Health didn’t mean that they actually had to give up any of their own policy space”* (Research workshop 2, health sector).

This co-benefits approach was described as a defining feature of the SA HiAP model and is consistent with the co-operation strategy described by Ollila.^42^ The actors implementing the HiAP approach did not always pursue areas with the clearest health benefits, but instead focussed primarily on securing the co-operation of other sectors. This involved offering health expertise to assist in addressing areas of interest/concern within other sectors, and finding ways to promote health while working on those areas.

### Strategies and Implementation

Three overarching *strategies* (mentioned at the beginning of the Results section above) emerged as fundamental to the SA HiAP initiative: the development of relational networks that connect individuals, agencies and sectors (this view was consistent with advocacy coalition theory^48^ in that it stresses the importance of interaction between policy actors in bringing about policy change); joint problem and opportunity identification and decision-making; and use of governance systems that connect HiAP with senior decision-makers.

The *implementation* of these strategies was seen as mediated by the culture, capacity and priorities of the agencies and sectors involved in those activities.

“*I think sometimes it might be about who’s around the table in the project and how empowered they feel in representing their organisation and also what the decision-making processes are in that organisation”* (Research workshop 1, non-health sector).

Power differentials and relationships between individual policy actors and agencies were identified as important factors shaping implementation.

“*I think there’s something that’s usually less stated than that, which is the power relationships … perceptions of different agencies around where they sit in the hierarchy and therefore whether they’ll actively contribute or whether they might sit on their hands a bit and pretend to be there”* (Research workshop 1, non-health sector).

*“… there were a lot of agencies around the table, there was clear jockeying and power differentials”* (Research workshop 2, health sector).

One participant noted that despite an emphasis on evidence-based policy, power could still trump evidence.

“…*people in powerful positions with personal opinions about what good ideas are, that don’t have an evidence base, but they get more traction because they’re in powerful positions”* (Research workshop 1, non-health sector).

Political will and resources available were also identified as key mediating factors in the implementation of the HiAP approach:

“…*can be hard to get your priorities from a Health in All Policy perspective into the priorities of the organisation. They might have other priorities …They don’t want to put everything up in one go because the Minister won’t like that…I also think the other thing is the actual cutbacks right across the government sector”* (Research workshop 1, non-health sector).

### Activities

The activities depicted in the framework relate to the actors, structures and processes of HiAP in SA. Figure 1 provides a high level descriptor for each of the key activities. Table 1 provides a précis of the activities under each of these descriptors through a selection of relevant quotes from the research workshops, and a summary of the role or mechanism that each of the activities fulfils.

**Table 1:** HiAP Activities and Associated Roles

**Activities and Pertinent Participant Quotes**	**Mechanisms**
Policy entrepreneurs *The Thinker in Residence and the importance of that because at that time we were also hosting Fred Hansen’s residency … So the notion of risk taking, if you like, was there in communicating across partners… across government through the Thinkers in Residency program* (Research workshop 1).	– Promote the potential and visibility of HiAP – Promote the benefits of inter-sectoral action Revitalise initiative – Maintain HiAP on policy agenda
Intermediaries *I think the key thing that got the ball rolling was that I had a supportive mindset of the people that I worked with but I guess it’s also that trust they had in me as well, that ‘if you think this is worth pursuing we’ll give you a go, see where it goes’* (Research workshop 1). *I think there was some of the people who – it resonated with them, that there was this relationship between the work and wellbeing* (Research workshop 2).	– Participate in early adoption of HiAP – ‘Champion’ HiAP – Transport and transmit HiAP ideas and practices within and across sectors – Watch for windows of opportunity
Relationship building and maintenance *Another thing that I think was really helpful was the relationship building and by that you built credibility* (Research workshop 1). *They’re in there listening. They’re looking for the alignment, they’re not in there telling ‘this is what you need to be doing’* (Research workshop 1).	– Informal and formal discussions – Persistence in relationship building – Negotiation – Explore diversity and common ground
Dedicated HiAP unit *I don’t think any of this would have happened unless there was a dedicated unit and the resources to go with it* (Research workshop 1). *It’s the competence of those that are actually trying to implement the theory … So it’s the fact it’s a dedicated resource and the nature of that resource that’s really important* (Research workshop 1). *You do need somebody, or a set of people working in the space that has an in depth understanding around determinants, public health, interventions, programs, how to create change, that new public health, all of that stuff* (Research workshop 2).	– Raises awareness – Mobilises stakeholders and resources – Negotiates roles, expectations – Provides leadership – ‘Shepherds’ participants – Facilitates creation of shared goals and co-benefits – Brings health expertise and skills in inter-sectoral collaboration – Watch for and use windows of opportunity – Develops understanding of partners’ agenda and core business
Health lens analysis *They were pretty rigorous in their statistical analysis, they were thorough in their research. They were empathetic in the way that they engaged. They were good at brokering and facilitating with other groups that we needed to work with* (Research workshop 1). *I think it’s important that there’s an understanding of what their policy area is in terms of its linkages to the social determinants of health and wellbeing* (Research workshop 2).	– Sharing information – Identify opportunities for cooperation – Articulate links between health lens analysis focus and health outcomes – Gather evidence – Explore options – Plan policy action
Central mandate for action *It does need that high level backing though, doesn’t it, because otherwise it ends up being the people who are converted already?* (Research workshop 1). *SASP [the SA Strategic Plan] was already there and all you needed to do was put a health lens over it and you know you’re ahead of the field* (Research workshop 2).	– Link HiAP work to existing policy framework and government priorities – Supports entry of Health into other sectors – Provides legitimation for Health and partners to act
Other HiAP initiatives S*eeing the opportunity through communications that HiAP had put out … overtly stating they wanted relationships with local government and seeing the opportunity* (Research workshop 1).	– Acts to promote awareness of HiAP principles and extend considerations of health and equity into other sectors – ‘Health in Planning’ initiative – Local Government engagement
Accountability and reporting *One of the things in favour of participating in the HiAP project was the fact that it was at that point reporting up to a body that I thought would have some influence for change* (Research workshop 1). *One of the other things that I think really helped us was the Eat Well Be Active Strategy and that that actually had a section at the back that said what [Department of Planning] had committed to, so that was I think a real achievement from Health’s perspective* (Research workshop 1).	– Link HiAP work to existing process for other cross government initiatives – Briefing of senior management – Chief Executive approval of proposals for HLA and recommendations from HLA – Legislative requirement eg, SA Public Health Act – Various State level health promotion and disease prevention plans

Abbreviations: HiAP‏, Health in All Policies; HLA, health lens analysis; SA, South Australia.

### Outputs and Outcomes

Outputs refer to the tangible products of the HiAP work such as literature reviews, reports, resources and new or amended policy or legislation. The intended ‘within government’ impacts and outputs of the HiAP activities were identified both during analysis of the documents and in the workshop data. The intermediate and longer term outcomes were also identified during the document analysis and workshops, and are expressed in general terms in the framework. These intermediate impacts on the policy environment, for example the creation of new alliances or convergence of agendas, were later explored in interviews and online surveys of public servants. Whether and how these impacts were realised during particular HLA projects was explored through case studies. The framework can incorporate evidence that is used to confirm or refute HiAP’s contribution, as is illustrated in the example below.

The ultimate goal of the HiAP intervention was the subject of considerable debate within and following the workshops. Early drafts of the framework posited “increased population health and health equity” as the ultimate goal. A number of workshop participants suggested this goal did not reflect non-health sectors’ objectives or the aim of achieving co-benefits. The final version of the framework incorporates concerns larger than health, phrased as: “SA Government’s goal of making SA a better place to live with increased population health and equity.”

#### Example

A HiAP HLA project to support healthy eating and increase physical activity identified 35 specific policy recommendations for action in 10 SA Government departments, which were subsequently incorporated in the State’s Eat Well Be Active Strategy 2011-2016.^49^ The Planning and Transport Department committed to 8 actions, one being to increase “Active Transport and public transport.” Implementation is demonstrated by completion of dedicated walking and cycling routes; bike boulevards which provide a low-speed environment on low-traffic streets for cyclists; and changes to Road Traffic Regulations which removed restrictions on riding on footpaths and widened the defined overtaking space for cars overtaking bicycles. These constitute an ‘investment in social determinants’ (see Outcomes section in Figure 1) and ‘within Government’ changes as represented in the framework. However, tracking the outcomes of these changes is necessary to consider whether and how these changes lead to the desired long-term outcomes, that is, to make SA a better place to live with increased population health and wellbeing. Progress of the Eat Well Be Active Strategy, including implementation of the cross government recommendations, will be reported to the South Australian Parliament.

The framework shown in Figure 1 is a representation of SA’s HiAP program theory, including a theory of change. The framework illustrates the processes that drive change and how they are understood to contribute to intended outcomes. It also includes the activities, such as the structures (eg, the HiAP unit) and processes (eg, the HLA) that have been constructed to “activate the theory of change.”^20^

Like all aspects of the framework, the outputs and outcomes sections were revised regularly as HiAP research monitored the activities and changing context and modes of implementation. The framework was adapted to reflect changes and developments as they occurred. The development of multiple iterations of the framework ensures its continued relevance and its ongoing utility as an evaluation tool.

## Discussion: How the Framework Has Guided Evaluation

Development of a program theory for the SA HiAP initiative provides a basis for evaluation in a number of ways. The iterative process and interaction between policy stakeholders and the research team provided an agreed framework to identify the aspects of HiAP to be monitored and evaluated and informed collection of empirical evidence to test and validate the program theory. It also provided a framework for the application of social and political science theory to the emerging evaluation results. Noordegraaf suggests that academic accounts of ‘real’ policy work are scarce - “how policy work is done, what acts and experiences contribute to what we see as policy, how bundles of acts and experiences make up policy dynamics, and how this might affect society.’^50^ As we open up the “black box” of policy-making through our action research, we are using relevant theories drawn from social and political science to interpret our results and test and understand the program theory (see Table 2). Examining the components and linkages between them has drawn our attention to particular questions regarding the operation of HiAP.

**Table 2:** Examples of How the Application of Social and Political Science Theory Informed the Evaluation

**Evaluation Questions**	**Theory**	**Focus**
• Why was HiAP successful in reaching the government agenda? • Why did particular HiAP projects succeed in reaching the agendas of multiple government departments? • How did HiAP continually realign to accommodate changing government agendas?	Agenda setting theory (Kingdon)^57^	Proposes that policy action requires a policy window to become available through linkage of problems, policy and politics.
• Why and how did other agencies and departments engage with HiAP? • What was the role of actors, such as champions, in diffusing HiAP ideas across sectors? • How did the political and bureaucratic systems support or impede HiAP’s focus on health equity and why did equity fail to get on the government agenda or drop from it?	Institutional theory (Howlett et al)^53^	Describes influence of actors, ideas and institutions in increasing acceptability of particular initiatives.
• How can expectations of competence and goodwill inform new inter-sectoral relationships? How is trust lost in inter-sectoral relationships and what are the impacts?	Trust theory (Giddens)^58,59^	Explains how trust serves to bridge the gap between the known and unknown in non-traditional ways of working, such as inter-sectoral action, to facilitate effective relationships.
• Why and how do particular HiAP activities and outputs lead to distal health outcomes for the SA population? • How can inter-sectoral action influence health and why is an inter-sectoral approach important in addressing health and its distribution (health equity)?	Social determinants of health theory (Solar and Irwin )^60^	Prompts analysis of the impacts of upstream distal factors on health and wellbeing.
• How does advocacy operate to strengthen or marginalise health promotion as a priority in health policy? • How do relational networks and the interactions of policy actors support policy change across sectors?	Advocacy Coalition Theory (Sabatier)^48^	Describes interaction between policy actors in bringing about policy change.
• How have different types of learning occurred during the development and implementation of HiAP? - instrumental learning, conceptual learning and social learning. • What is the nature of the learning that has occurred within the government and what impacts has learning produced on policy and practices?	Policy networks and policy learning (Sabatier)^51^	Considers how learning occurs over time within policy networks and how it produces changes in values, goals, processes and meanings.

Abbreviations: HiAP‏, Health in All Policies; SA, South Australia.

For example, we are drawing on the advocacy coalition framework in our analysis of how non-health sectors have taken-up and experienced HiAP.^51-53^ Institutional theory is informing our data collection and analysis of the role of institutions, actors and ideas in HiAP.^53-55^ Our analysis is also drawing on constructs from theories of policy networks and policy learning.^48,53,56^

The testing and re-testing of the program theory has occurred on an ongoing basis and the results of our research have continued to refine this theory. Part of the evidence considered was concerned with implementation in order to determine whether the various HiAP components have been implemented as predicted by the framework. This means determining that “real activities in action and not just espoused ideas of activities” (p. 427) are in fact enacting the program theory.^38^ For example, the role of particular actors, ‘policy entrepreneurs,’ was theorised to be a means of promoting the benefits of HiAP and maintaining it on the government agenda. In Kingdon’s model, policy entrepreneurs may act to bring the three policy system streams together creating a policy window of opportunity.^57^ Evidence then was sought to validate the existence and theorised impacts of policy entrepreneurs.

**Figure 2:**
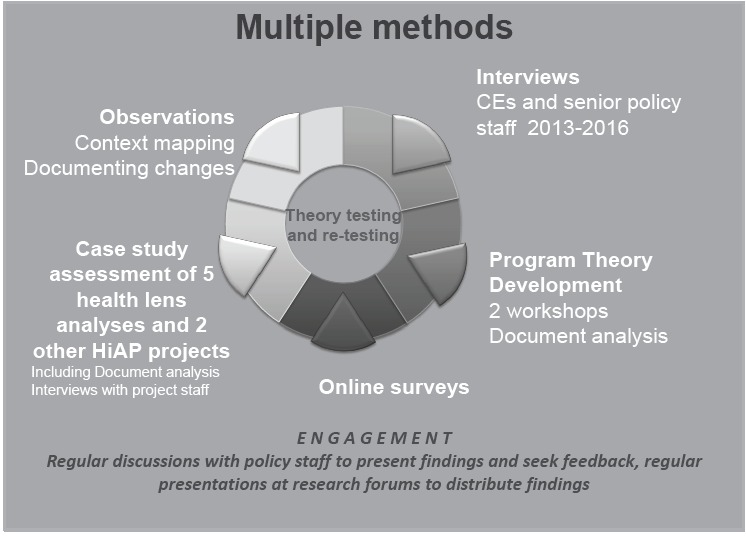
Multiple Methods of Data Collection.

Theory based evaluation is not prescriptive of methods to be used, rather it enables a range of methods to be employed and multiple data sources used to construct and test program theory (see Figure 2). The document analysis and workshops rendered activities visible and provided the basis for them to be examined more closely. In line with recommendations regarding ‘getting close’ to policy participants, we undertook in-depth interviews with key informants which further explored how HiAP operates and how the activities involved may facilitate and/or impede aspects of HiAP work.^61^ Repeated rounds of interviews allowed us to capture change over time and in context. Five case studies of Health Lens Analyses allowed cross-case comparisons to determine how the same strategy or activity played out in different contexts and shaped outcomes. This allowed inferences to be drawn regarding the interaction of context with the initiative.

Taking account of context also directed the researchers’ attention to studying and understanding how and why the HiAP initiative changed over time in response to broader circumstances and how policy actors negotiated the changes. For example, during the course of the research, a perception of State economic instability and contraction emerged in SA. This perception was met with a decision to cut the public service, and to apply only a narrow health agenda, focussed predominantly on the provision of curative medical services. The retreat from health promotion activities resulted in the discontinuation of several, long-standing health promotion activities that previously operated as ‘fellow travellers’ for HiAP, including the abandonment of the State’s Primary Prevention Plan. While tracking such developments, the researchers ensured that the contextual changes were reflected in revised versions of the framework. Institutional theory was applied to assist in understanding the Government’s response,^54,55^ and its potential implications for HiAP. Application of the theory encouraged the researchers to consider the interactions between institutions, actors and ideas. Empirical data was analysed to reveal the ways that HiAP actors coped with the institutional response, and to identify changes in the ways that HiAP was being implemented to ensure its survival in an increasingly hostile environment that did not favour health promotion. This analysis resulted in a deepening understanding about the barriers and facilitators of HiAP within its Government context, and illuminated links between the strategies involved in implementing HiAP, the associated activities of actors and the success of HiAP in being able to achieve sufficient ongoing support within the government environment (impacts column in Figure 1).

The framework also shaped collection of evidence regarding the short term and intermediate changes, and, where possible, longer term outcomes. The achievement of the ‘big picture’ outcomes is predicated on the achievement of the activities and outputs that the framework suggests are required. Where empirical evidence can be found for achievement of the links in the chain “then it can be reasonably predicted that the outcomes are at least in part attributable to the program.”^62^ Mayne^18^ also notes that to strengthen the analysis of causality the various assumptions in the theory of change must also be tested for validity. Do the results of our study support the assumptions? Is there other evidence to support the assumptions? The role of other external influencing factors must also be considered. The aim of evidence-gathering and analysis is to “reduce uncertainty about the contribution an intervention is making to observed results through an increased understanding of why results did or did not occur and the roles played by the intervention and other influencing factors” (p. 271).^18^ These questions and considerations were reflected on during revisions of the framework.

Examination of the interactions between various components of the framework allowed us to ask what kinds of factors are likely to lead to ‘success’ or ‘disappointment’ in terms of the observed or predicted outcomes.^63^ The complexity of policy-making and implementation can be subject to “negotiation, resistance, adaptation, leak and borrow, bloom and fade,” all of which can shape processes and affect outcomes.^64^

The framework has provided a means of tracking changes to the model of HiAP and the program theory inherent in it. The assumptions underpinning the model are being tested against the emergent data at several points during the research and frequently revised to ensure that the assumptions accurately reflected the ideas that are emerging. The South Australian model altered in response to changing policy priorities. For example, the structural organisation of HiAP around a “dedicated HiAP unit” was originally theorised as a key component of the framework. Post 2013 an organisational restructure led to the integration of the unit into a group with a wider public health remit and a change in their activities. This provided the researchers with an opportunity to examine a challenge to the program theory and the ways in which this change manifested itself in terms of policy-making processes and the actions that arose from them. Subsequent papers will present the detailed findings from the evaluation which will be shaped by the framework presented in this paper.

### Strengths

Our evaluation approach used a mix of theoretical knowledge from social and political science domains combined with practice wisdom in the co-design process. Stakeholders were engaged in development of the framework, thus strengthening the research–policy relationships required to make the study feasible and allowing researchers to get close to policy work. The framework provided a means of describing the HiAP initiative at a point in time and, through ongoing testing of the framework, data collection and theory testing, allowed developments to be captured and reflected in subsequent models. It provided a framework to guide data collection, which attempted to take account of the complexity of policy work and context, and the design of HiAP. The framework allowed assessment of outcomes through a logic process that used prediction and burden of evidence as a base for claiming outcomes.

## Limitations

This paper presents the framework for the evaluation and gives examples of its use, but does not present evaluation results, which have been,^40,61^ and will be, presented elsewhere.

The framework developed in 2013 was static and represented a point in time and so has required updating as the research has progressed. Our ability to update and develop several iterations has helped to provide graphical representations of how the policy initiative has evolved over time. As such, the revision process facilitated deep understanding about how and why the initiative changed. There is inevitably some tension between capturing local knowledge and experience and eliminating bias, which calls for particular attention in our research to triangulating data sources and seeking disconfirming accounts. The process is time-consuming and requires high levels of reflexivity in both the research team and the research participants. The work is not generalizable in the sense it is proposed in quantitative research, but it can provide working hypotheses that may be used to understand other cases and contexts.^65,66^

## Conclusion

Theory-based evaluation provides a means of examining the processes involved in policy-making and makes an assessment of the contribution of policy interventions to distal policy outcomes. In the case of HiAP the development of a theory-based framework responded to calls for research on cross government mechanisms to support health and equity and establishes a framework for an empirical study of the SA Government HiAP approach. The process of developing the framework was collaborative and iterative and engaged researchers and policy actors in articulating the underlying theory of the South Australian HiAP initiative. The method outlined in this paper provides a means for developing a rigorous evaluation framework that articulates the causal chains underpinning the anticipated short, intermediate and longer term impacts of the HiAP approach and resulting policy. As such the method lays the foundations for explaining the pathways through which the health impacts of policy may be realised, even if policy research projects cannot continue long enough to see these impacts eventuate.

## Acknowledgements

We acknowledge the input of other Chief and Associate Investigators who contributed to the design of this research: Jennie Popay, Michael Marmot, Elizabeth Harris, Danny Broderick, Ilona Kickbusch, Kevin Buckett, Sandy Pitcher, Andrew Stanley, and Deborah Wildgoose.

This work was supported by the National Health and Medical Research Council (grant number 1027561).

The views expressed in this paper do not necessarily reflect those of the South Australian Government.

## Ethical issues

All data collection activities received prior approval from the Flinders University Ethics Committee (Project 5518) and the SA Health Ethics Committee (Project HREC/12/SAH/74). All participants were provided with an information sheet regarding the project and signed a consent form, including consent to publish.

## Competing interests

CW is the Manager, Health Determinants and Policy, Department of Health and Ageing with responsibility for Health in All Policies work. The other authors have no competing interests.

## Authors’ contributions

AL wrote and revised the paper, contributed to conceptual design of the study and the collection, analysis and interpretation of data. FB led the conceptual design of the study, contributed to the collection, analysis and interpretation of data, and provided comment on drafts. TDC contributed to the conceptual design of the study, collection, analysis and interpretation of data, provided comment on drafts. CMcD contributed to conceptual design of the study, the collection, analysis and interpretation of data, and provided comment on drafts.

CW contributed to conceptual design of the study and interpretation of data and provided comment on drafts. DMcD contributed to conceptual design of the study and the collection, analysis and interpretation of data and provided comment on drafts. HvE contributed to the analysis and interpretation of data, and provided comment on drafts. All authors read and approved the final manuscript.

## Authors’ affiliations

^1^College of Nursing and Health Sciences, Flinders University, Adelaide, SA, Australia. ^2^Southgate Institute for Health Society and Equity, Flinders University, Adelaide, SA, Australia. ^3^College of Medicine and Public Health, Flinders University, Adelaide, SA, Australia. ^4^Department of Health and Ageing, Adelaide, SA, Australia. ^5^The Poche Centre for Indigenous Health and Well-being, Flinders University, Adelaide, SA, Australia.

## Key messages

**Implications for policy makers**

- Program theory-based evaluation provides an approach to evaluate the process and longer term outcomes (through a predictive chain-of-logic approach) of complex inter-sectoral policy processes.
- Developing the evaluation framework through a participatory and iterative process enables the involvement of policy actors and facilitates co-production of knowledge.
- This approach to evaluation can account for the changing political and bureaucratic environments that are part of the reality of policy-making.
- Program theory-based evaluation is currently the best approach to determine, prospectively, the health impacts of policy.

**Implications for the public**

Public policy shapes the social, economic and environmental conditions of everyday living. These conditions influence the health and wellbeing of individuals and populations. Action to improve health requires multiple policy sectors and policy-makers to work together to achieve improved health and ensure that benefits are distributed equitably across the population. Despite the importance of such inter-sectoral work it has been difficult to evaluate given the complexity of the task, the wide range of sectors and people involved, and the difficulties of attributing long-term outcomes to policy changes. This paper describes development of a framework for evaluation that allows examination of both the policy-making processes and the health outcomes of the resulting policies.
